# 6-Chloro-3-[(di­methyl­amino)­methyl­idene]thio­chroman-4-one

**DOI:** 10.1107/S1600536813024550

**Published:** 2013-09-07

**Authors:** Ashraf Y. Khan, Nikhath Fathima, Mallikarjun B. Kalashetti, Noor Shahina Begum, I. M. Khazi

**Affiliations:** a Department of Chemistry, Karnatak University, Dharwad 580 003, India; b Department of Studies in Chemistry, Bangalore University, Bangalore 560 001, Karnataka, India

## Abstract

The asymmetric unit of the title compound, C_12_H_12_ClNOS, contains three independent mol­ecules, with the thio­chroman ring adopting a sofa conformation in each one. The crystal structure features C—H⋯O inter­actions; one of the O atoms accepts three such bonds. Together, the hydrogen bonds give rise to a molecular tape propagating in [010].

## Related literature

For general background and the anti­fungal activity of thio­chromans, see: Wang *et al.* (2010 ▶); Sosnovskikh (2003 ▶). For the crystal structure of a related compound, see: Butt *et al.* (1988 ▶).
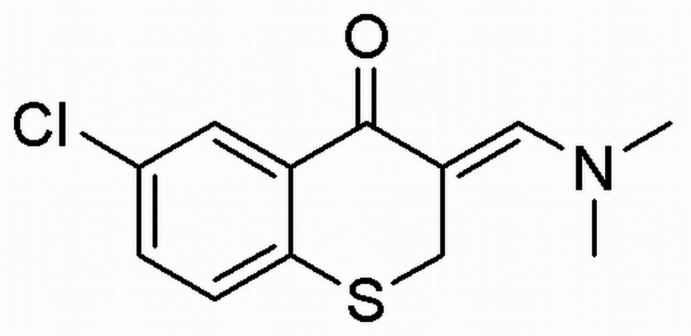


## Experimental

### 

#### Crystal data

C_12_H_12_ClNOS
*M* *_r_* = 253.74
Monoclinic, 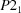

*a* = 11.0031 (3) Å
*b* = 12.5937 (3) Å
*c* = 13.0787 (3) Å
β = 100.255 (2)°
*V* = 1783.36 (8) Å^3^
*Z* = 6
Mo *K*α radiation
μ = 0.47 mm^−1^
*T* = 296 K
0.18 × 0.16 × 0.16 mm

#### Data collection

Bruker SMART APEX CCD detector diffractometer
Absorption correction: multi-scan (*SADABS*; Bruker, 1998 ▶) *T*_min_ = 0.920, *T*_max_ = 0.928
13306 measured reflections
6540 independent reflections
5418 reflections with *I* > 2σ(*I*)
*R*_int_ = 0.023

#### Refinement

*R*[*F* ^2^ > 2σ(*F* ^2^)] = 0.039
*wR*(*F* ^2^) = 0.087
*S* = 1.01
6540 reflections
440 parameters
1 restraint
H-atom parameters constrained
Δρ_max_ = 0.20 e Å^−3^
Δρ_min_ = −0.32 e Å^−3^

### 

Data collection: *SMART* (Bruker, 1998 ▶); cell refinement: *SAINT-Plus* (Bruker, 1998 ▶); data reduction: *SAINT-Plus*; program(s) used to solve structure: *SHELXS97* (Sheldrick, 2008 ▶); program(s) used to refine structure: *SHELXL97* (Sheldrick, 2008 ▶); molecular graphics: *ORTEP-3* (Farrugia, 2012 ▶) and *CAMERON* (Watkin *et al.*, 1996) ▶; software used to prepare material for publication: *WinGX* (Farrugia, 2012 ▶).

## Supplementary Material

Crystal structure: contains datablock(s) global, I. DOI: 10.1107/S1600536813024550/pv2641sup1.cif

Structure factors: contains datablock(s) I. DOI: 10.1107/S1600536813024550/pv2641Isup2.hkl

Supplementary material file. DOI: 10.1107/S1600536813024550/pv2641Isup3.cml

Additional supplementary materials:  crystallographic information; 3D view; checkCIF report

## Figures and Tables

**Table 1:** Hydrogen-bond geometry (Å, °)

*D*—H⋯*A*	*D*—H	H⋯*A*	*D*⋯*A*	*D*—H⋯*A*
C1a—H1a2⋯O1b^i^	0.97	2.39	3.235 (4)	144
C11a—H11d⋯O1b^i^	0.96	2.63	3.290 (4)	126
C1b—H1b2⋯O1a	0.97	2.43	3.284 (1)	147
C12a—H12b⋯O1b	0.96	2.59	3.358 (4)	137

Symmetry code: (i) 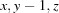.

**Fig. 1.**
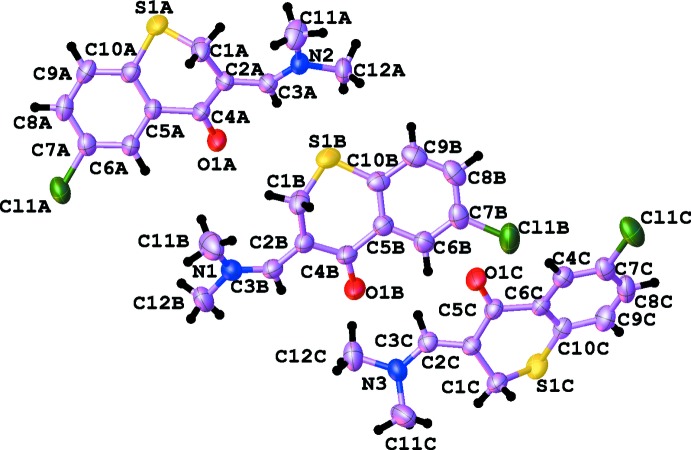
ORTEP (Farrugia, 2012) diagram of the three independent molecules present in the asymmetric unit of the title compound, showing 50% probability displacement ellipsoids and the atom-numbering scheme.

**Fig. 2.**
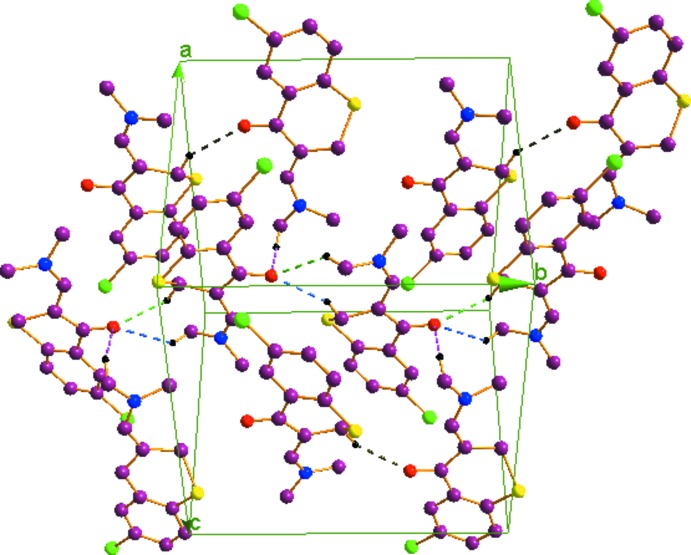
A unit cell packing of the title compound showing intermolecular interactions with dotted lines. H-atoms not involved in hydrogen bonding have been excluded.

## supplementary crystallographic information

### 1. Comment

Thiochromanones belong to an important class of oxygen containing heterocycles; many of their derivatives have been reported to possess important biological activities including antifungal activity (Wang *et al.*,2010). They also serve as the starting material for the synthesis of novel heterocyclic systems (Sosnovskikh, 2003).

There are three crystallographically independent molecules (A, B and C) in an asymmetric unit of the title compond (Fig. 1) wherein 6-chloro-thiochroman moiety is substituted with the dimethylaminomethylene group at C2. The dimethylamino group is oriented *trans* with respect to the oxo group of the thiochroman moiety which is described by the torsion angles N1—C3A—C2A –C4A, N2—C3B—C2B—C4B and N3—C3C—C2C—C4C [172.25 (3), -173.45 (2) and -171.53 (3)°] for the molecules A, B and C, respectively. The thiochroman rings in the three molecules are significantly puckered and adopt sofa conformations. A mean-planes calculation shows that the atoms S1A, S1B and S1C deviate from the mean planes of the remaining ring atoms by 0.7536 (1), -0.7360 (1) and -0.6753 (1) Å, respectively. The bond distances and angles in the three molecules of the title compound agree very well with the corresponding bond distances and angles reported in a closely related compound (Butt *et al.*, 1988).

The crystal structure is stabilized by C—H···O type intermolecular interactions (Tab. 1 & Fig. 2); three such interactions form trifurcated bonds from three donors C1A, C11A and C12A to the same acceptor O1B, linking the molecules in a tape like structure. Whereas, another C—H···O interaction results in a one dimensional chain along the *b*-axis.

### 2. Experimental

A mixture of 6-chloro -thiochroman-4-one (0.01 mol) and dimethylformamide-dimethylacetal (DMF-DMA) (2 mL) was heated under reflux for 10 h. The reaction mixture was triturated with ethanol to give a solid product that was collected by filtration and crystallized from ethanol to give the title compound as deep yellow crystals, melting point 379–381 K. Yield 78%.

### 3. Refinement

The H atoms were placed at calculated positions in the riding model approximation with C—H = 0.97° A, 0.93 Å and 0.96 Å for aromatic, heterocyclic and methyl H-atoms respectively, with *U*_iso_(H) = 1.5*U*_eq_(C) for methyl H atoms and *U*_iso_(H) = 1.2*U*_eq_(N/C). Since the crystals contained racemic twins, an absolute structure could not be established and therefore, 2865 Friedel pairs of reflections were merged.

### Crystal data

**Table:**

C_12_H_12_ClNOS	*F*(000) = 792
*M**_r_* = 253.74	*D*_x_ = 1.418 Mg m^−^^3^
Monoclinic, *P*2_1_	Mo *K*α radiation, λ = 0.71073 Å
Hall symbol: P 2yb	Cell parameters from 6540 reflections
*a* = 11.0031 (3) Å	θ = 1.6–26.1°
*b* = 12.5937 (3) Å	µ = 0.47 mm^−^^1^
*c* = 13.0787 (3) Å	*T* = 296 K
β = 100.255 (2)°	Block, yellow
*V* = 1783.36 (8) Å^3^	0.18 × 0.16 × 0.16 mm
*Z* = 6	

### Data collection

**Table:**

Bruker SMART APEX CCD detector diffractometer	6540 independent reflections
Radiation source: fine-focus sealed tube	5418 reflections with *I* > 2σ(*I*)
Graphite monochromator	*R*_int_ = 0.023
ω scans	θ_max_ = 26.1°, θ_min_ = 1.6°
Absorption correction: multi-scan (*SADABS*; Bruker, 1998)	*h* = −13→11
*T*_min_ = 0.920, *T*_max_ = 0.928	*k* = −15→15
13306 measured reflections	*l* = −15→16

### Refinement

**Table:**

Refinement on *F*^2^	Primary atom site location: structure-invariant direct methods
Least-squares matrix: full	Secondary atom site location: difference Fourier map
*R*[*F*^2^ > 2σ(*F*^2^)] = 0.039	Hydrogen site location: inferred from neighbouring sites
*wR*(*F*^2^) = 0.087	H-atom parameters constrained
*S* = 1.01	*w* = 1/[σ^2^(*F*_o_^2^) + (0.0363*P*)^2^ + 0.3396*P*] where *P* = (*F*_o_^2^ + 2*F*_c_^2^)/3
6540 reflections	(Δ/σ)_max_ = 0.001
440 parameters	Δρ_max_ = 0.20 e Å^−^^3^
1 restraint	Δρ_min_ = −0.32 e Å^−^^3^

### Special details

**Table:**

Geometry. All s.u.'s (except the s.u. in the dihedral angle between two l.s. planes) are estimated using the full covariance matrix. The cell s.u.'s are taken into account individually in the estimation of s.u.'s in distances, angles and torsion angles; correlations between s.u.'s in cell parameters are only used when they are defined by crystal symmetry. An approximate (isotropic) treatment of cell s.u.'s is used for estimating s.u.'s involving l.s. planes.
Refinement. Refinement of *F*^2^ against ALL reflections. The weighted *R*-factor *wR* and goodness of fit *S* are based on *F*^2^, conventional *R*-factors *R* are based on *F*, with *F* set to zero for negative *F*^2^. The threshold expression of *F*^2^ > 2σ(*F*^2^) is used only for calculating *R*-factors(gt) *etc*. and is not relevant to the choice of reflections for refinement. *R*-factors based on *F*^2^ are statistically about twice as large as those based on *F*, and *R*- factors based on ALL data will be even larger.

### Fractional atomic coordinates and isotropic or equivalent isotropic displacement parameters (Å^2^)

**Table:**

	*x*	*y*	*z*	*U*_iso_*/*U*_eq_	
S1C	−3.42533 (9)	−0.93986 (6)	−0.35478 (6)	0.0616 (2)	
S1B	−3.07997 (9)	−1.52311 (6)	−0.62968 (7)	0.0660 (2)	
Cl1C	−3.52910 (9)	−1.26958 (9)	−0.02286 (7)	0.0825 (3)	
S1A	−3.07889 (9)	−2.03016 (7)	−0.89801 (6)	0.0696 (3)	
Cl1B	−3.19333 (10)	−1.20168 (9)	−0.29486 (7)	0.0839 (3)	
C6B	−3.1874 (3)	−1.2629 (3)	−0.4910 (2)	0.0522 (8)	
H6B	−3.2190	−1.1957	−0.5095	0.063*	
C4C	−3.5168 (3)	−1.1744 (2)	−0.4045 (2)	0.0437 (7)	
O1C	−3.5194 (2)	−1.27163 (15)	−0.41754 (16)	0.0634 (6)	
C6C	−3.5265 (3)	−1.2055 (3)	−0.2193 (2)	0.0481 (7)	
H6C	−3.5614	−1.2713	−0.2389	0.058*	
O1A	−3.1373 (2)	−1.69412 (16)	−0.86749 (15)	0.0683 (7)	
N1	−3.2097 (2)	−1.3641 (2)	−0.95226 (19)	0.0548 (6)	
C5B	−3.1633 (3)	−1.3321 (2)	−0.5676 (2)	0.0469 (7)	
C2C	−3.5249 (3)	−1.0987 (2)	−0.4879 (2)	0.0417 (7)	
C5C	−3.5017 (2)	−1.1352 (2)	−0.2945 (2)	0.0401 (6)	
C2B	−3.1897 (3)	−1.3663 (2)	−0.7622 (2)	0.0480 (7)	
N2	−3.1930 (2)	−1.85555 (19)	−0.61376 (18)	0.0487 (6)	
C6A	−3.1601 (3)	−1.7658 (2)	−1.0679 (2)	0.0470 (7)	
H6A	−3.1830	−1.6959	−1.0582	0.056*	
C3B	−3.1897 (3)	−1.3229 (2)	−0.8579 (2)	0.0486 (7)	
H3B	−3.1724	−1.2506	−0.8563	0.058*	
N3	−3.5292 (2)	−1.0987 (2)	−0.67712 (19)	0.0542 (7)	
C5A	−3.1416 (3)	−1.8353 (2)	−0.9845 (2)	0.0429 (7)	
C9B	−3.0975 (3)	−1.4623 (3)	−0.4334 (3)	0.0611 (9)	
H9B	−3.0674	−1.5297	−0.4137	0.073*	
C10C	−3.4542 (3)	−1.0344 (2)	−0.2642 (2)	0.0455 (7)	
C3C	−3.5134 (3)	−1.1407 (2)	−0.5828 (2)	0.0455 (7)	
H3C	−3.4902	−1.2118	−0.5803	0.055*	
C3A	−3.1682 (3)	−1.8183 (2)	−0.7022 (2)	0.0449 (7)	
H3A	−3.1447	−1.7473	−0.6987	0.054*	
C4A	−3.1493 (3)	−1.7912 (2)	−0.8782 (2)	0.0465 (7)	
O1B	−3.1807 (3)	−1.19390 (17)	−0.68895 (17)	0.0804 (8)	
C2A	−3.1701 (3)	−1.8629 (2)	−0.7978 (2)	0.0454 (7)	
C10B	−3.1184 (3)	−1.4340 (2)	−0.5381 (2)	0.0494 (7)	
C8B	−3.1206 (3)	−1.3923 (3)	−0.3592 (3)	0.0618 (9)	
H8B	−3.1063	−1.4118	−0.2895	0.074*	
C4B	−3.1791 (3)	−1.2914 (2)	−0.6772 (2)	0.0515 (8)	
C9C	−3.4249 (3)	−1.0108 (3)	−0.1581 (2)	0.0600 (9)	
H9C	−3.3896	−0.9455	−0.1371	0.072*	
C12A	−3.1723 (3)	−1.7894 (3)	−0.5204 (2)	0.0600 (9)	
H12A	−3.1380	−1.7224	−0.5358	0.090*	
H12B	−3.1160	−1.8246	−0.4665	0.090*	
H12C	−3.2494	−1.7776	−0.4975	0.090*	
C10A	−3.1085 (3)	−1.9398 (2)	−1.0013 (2)	0.0478 (7)	
C12B	−3.1897 (3)	−1.3005 (3)	−1.0410 (2)	0.0672 (10)	
H12D	−3.1612	−1.2311	−1.0176	0.101*	
H12E	−3.2659	−1.2943	−1.0895	0.101*	
H12F	−3.1289	−1.3343	−1.0743	0.101*	
C7A	−3.1449 (3)	−1.7990 (2)	−1.1649 (2)	0.0503 (7)	
C1B	−3.1963 (3)	−1.4825 (2)	−0.7383 (2)	0.0601 (9)	
H1B1	−3.1860	−1.5232	−0.7992	0.072*	
H1B2	−3.2773	−1.4986	−0.7230	0.072*	
C12C	−3.4992 (4)	−1.1612 (3)	−0.7633 (2)	0.0730 (11)	
H12G	−3.4688	−1.2296	−0.7383	0.109*	
H12H	−3.4371	−1.1251	−0.7932	0.109*	
H12I	−3.5721	−1.1701	−0.8152	0.109*	
C8C	−3.4473 (3)	−1.0822 (3)	−0.0849 (3)	0.0650 (10)	
H8C	−3.4273	−1.0656	−0.0146	0.078*	
C9A	−3.0919 (3)	−1.9717 (2)	−1.1001 (2)	0.0542 (8)	
H9A	−3.0677	−2.0411	−1.1104	0.065*	
C8A	−3.1110 (3)	−1.9021 (2)	−1.1819 (2)	0.0546 (8)	
H8A	−3.1011	−1.9240	−1.2479	0.066*	
C7B	−3.1653 (3)	−1.2924 (3)	−0.3887 (2)	0.0566 (8)	
C1C	−3.5392 (3)	−0.9836 (2)	−0.4629 (2)	0.0525 (8)	
H1C1	−3.6210	−0.9722	−0.4469	0.063*	
H1C2	−3.5322	−0.9412	−0.5235	0.063*	
C7C	−3.4997 (3)	−1.1785 (3)	−0.1159 (2)	0.0527 (8)	
C11B	−3.2578 (4)	−1.4694 (3)	−0.9776 (3)	0.0897 (13)	
H11A	−3.3090	−1.4903	−0.9288	0.134*	
H11B	−3.1906	−1.5185	−0.9745	0.134*	
H11C	−3.3060	−1.4693	−1.0465	0.134*	
C1A	−3.1902 (4)	−1.9785 (2)	−0.8248 (2)	0.0615 (9)	
H1A1	−3.2725	−1.9876	−0.8651	0.074*	
H1A2	−3.1854	−2.0191	−0.7613	0.074*	
C11A	−3.2427 (4)	−1.9601 (3)	−0.5984 (3)	0.0751 (10)	
H11D	−3.1774	−2.0116	−0.5904	0.113*	
H11E	−3.3043	−1.9783	−0.6574	0.113*	
H11F	−3.2794	−1.9595	−0.5370	0.113*	
C11C	−3.5908 (4)	−0.9984 (3)	−0.7056 (3)	0.0877 (13)	
H11G	−3.5308	−0.9424	−0.6989	0.131*	
H11H	−3.6490	−0.9846	−0.6606	0.131*	
H11I	−3.6335	−1.0020	−0.7763	0.131*	
Cl1A	−3.16764 (10)	−1.70872 (8)	−1.26739 (6)	0.0761 (3)	

### Atomic displacement parameters (Å^2^)

**Table:**

	*U*^11^	*U*^22^	*U*^33^	*U*^12^	*U*^13^	*U*^23^
S1C	0.0853 (6)	0.0400 (4)	0.0618 (5)	−0.0166 (4)	0.0197 (4)	−0.0109 (4)
S1B	0.0957 (7)	0.0386 (4)	0.0702 (5)	0.0110 (5)	0.0320 (5)	0.0116 (4)
Cl1C	0.0880 (7)	0.1115 (8)	0.0503 (5)	−0.0071 (6)	0.0187 (5)	0.0184 (5)
S1A	0.1128 (8)	0.0386 (4)	0.0568 (5)	0.0172 (5)	0.0133 (5)	−0.0039 (4)
Cl1B	0.0925 (7)	0.1090 (8)	0.0526 (5)	0.0087 (6)	0.0194 (5)	−0.0158 (5)
C6B	0.0514 (18)	0.0576 (19)	0.0489 (18)	0.0055 (15)	0.0125 (14)	0.0025 (15)
C4C	0.0505 (17)	0.0363 (16)	0.0447 (16)	−0.0032 (13)	0.0098 (14)	−0.0079 (13)
O1C	0.1068 (19)	0.0324 (12)	0.0515 (13)	−0.0062 (12)	0.0155 (12)	−0.0053 (10)
C6C	0.0482 (17)	0.0466 (17)	0.0499 (17)	−0.0011 (14)	0.0096 (14)	−0.0033 (15)
O1A	0.125 (2)	0.0362 (12)	0.0446 (12)	0.0043 (12)	0.0184 (13)	−0.0032 (10)
N1	0.0678 (17)	0.0477 (15)	0.0473 (15)	0.0021 (13)	0.0064 (13)	0.0003 (12)
C5B	0.0487 (17)	0.0460 (17)	0.0477 (17)	−0.0008 (13)	0.0134 (14)	0.0023 (14)
C2C	0.0479 (17)	0.0335 (15)	0.0431 (16)	−0.0022 (13)	0.0061 (13)	−0.0032 (12)
C5C	0.0400 (15)	0.0379 (15)	0.0436 (16)	0.0017 (13)	0.0102 (13)	−0.0043 (13)
C2B	0.0581 (19)	0.0429 (16)	0.0461 (17)	0.0021 (14)	0.0181 (15)	0.0002 (14)
N2	0.0630 (16)	0.0437 (13)	0.0413 (14)	−0.0006 (12)	0.0147 (12)	−0.0034 (11)
C6A	0.0568 (18)	0.0449 (16)	0.0393 (16)	0.0040 (14)	0.0087 (14)	−0.0046 (13)
C3B	0.0571 (19)	0.0392 (16)	0.0507 (18)	0.0042 (14)	0.0127 (15)	0.0006 (14)
N3	0.0687 (17)	0.0522 (16)	0.0400 (14)	−0.0030 (13)	0.0052 (12)	−0.0006 (12)
C5A	0.0489 (16)	0.0438 (16)	0.0354 (14)	0.0019 (13)	0.0056 (13)	−0.0064 (12)
C9B	0.061 (2)	0.059 (2)	0.063 (2)	−0.0057 (17)	0.0113 (17)	0.0163 (18)
C10C	0.0439 (16)	0.0463 (17)	0.0468 (16)	0.0012 (14)	0.0098 (13)	−0.0093 (14)
C3C	0.0500 (18)	0.0395 (16)	0.0466 (17)	−0.0057 (13)	0.0077 (14)	−0.0042 (13)
C3A	0.0538 (18)	0.0386 (15)	0.0415 (16)	0.0007 (13)	0.0062 (14)	−0.0019 (13)
C4A	0.063 (2)	0.0355 (16)	0.0392 (15)	0.0063 (14)	0.0040 (14)	−0.0059 (12)
O1B	0.152 (2)	0.0398 (13)	0.0529 (13)	0.0160 (14)	0.0292 (15)	0.0045 (11)
C2A	0.0602 (18)	0.0367 (15)	0.0373 (15)	0.0003 (14)	0.0028 (13)	−0.0036 (12)
C10B	0.0513 (17)	0.0404 (16)	0.0595 (18)	−0.0021 (14)	0.0181 (15)	0.0091 (14)
C8B	0.058 (2)	0.080 (3)	0.0480 (19)	−0.0077 (18)	0.0098 (16)	0.0125 (17)
C4B	0.068 (2)	0.0373 (17)	0.0514 (18)	0.0077 (14)	0.0155 (15)	0.0042 (13)
C9C	0.066 (2)	0.056 (2)	0.0575 (19)	−0.0087 (17)	0.0102 (16)	−0.0185 (17)
C12A	0.080 (2)	0.065 (2)	0.0366 (16)	0.0002 (18)	0.0161 (16)	−0.0089 (15)
C10A	0.0505 (17)	0.0430 (15)	0.0480 (16)	0.0014 (14)	0.0040 (13)	−0.0084 (14)
C12B	0.080 (2)	0.082 (2)	0.0405 (17)	0.008 (2)	0.0133 (17)	0.0061 (17)
C7A	0.0535 (18)	0.0584 (19)	0.0404 (15)	−0.0002 (15)	0.0124 (14)	−0.0010 (14)
C1B	0.089 (3)	0.0401 (16)	0.0564 (19)	−0.0102 (16)	0.0270 (18)	−0.0038 (14)
C12C	0.092 (3)	0.084 (3)	0.0468 (19)	−0.013 (2)	0.0222 (19)	−0.0105 (18)
C8C	0.066 (2)	0.089 (3)	0.0405 (17)	0.002 (2)	0.0099 (16)	−0.0137 (17)
C9A	0.0592 (19)	0.0485 (18)	0.0567 (19)	0.0008 (15)	0.0152 (16)	−0.0180 (15)
C8A	0.0575 (19)	0.064 (2)	0.0455 (18)	−0.0075 (16)	0.0185 (15)	−0.0153 (15)
C7B	0.0487 (18)	0.078 (2)	0.0445 (17)	−0.0041 (17)	0.0123 (14)	−0.0021 (17)
C1C	0.069 (2)	0.0399 (16)	0.0499 (17)	0.0041 (14)	0.0140 (15)	−0.0014 (13)
C7C	0.0477 (18)	0.068 (2)	0.0431 (17)	0.0013 (16)	0.0102 (14)	0.0022 (16)
C11B	0.131 (4)	0.069 (3)	0.061 (2)	−0.016 (2)	−0.005 (2)	−0.0070 (19)
C1A	0.102 (3)	0.0403 (16)	0.0446 (16)	−0.0135 (17)	0.0193 (17)	−0.0056 (14)
C11A	0.101 (3)	0.060 (2)	0.076 (2)	−0.025 (2)	0.047 (2)	−0.0089 (19)
C11C	0.123 (4)	0.071 (3)	0.057 (2)	0.016 (2)	−0.014 (2)	0.0074 (19)
Cl1A	0.1077 (7)	0.0810 (6)	0.0412 (4)	0.0031 (6)	0.0176 (4)	0.0045 (4)

### Geometric parameters (Å, º)

**Table:**

S1C—C10C	1.749 (3)	C10C—C9C	1.399 (4)
S1C—C1C	1.801 (3)	C3C—H3C	0.9300
S1B—C10B	1.748 (3)	C3A—C2A	1.367 (4)
S1B—C1B	1.808 (4)	C3A—H3A	0.9300
Cl1C—C7C	1.743 (3)	C4A—C2A	1.434 (4)
S1A—C10A	1.752 (3)	O1B—C4B	1.238 (3)
S1A—C1A	1.804 (3)	C2A—C1A	1.505 (4)
Cl1B—C7B	1.744 (3)	C8B—C7B	1.380 (4)
C6B—C7B	1.369 (4)	C8B—H8B	0.9300
C6B—C5B	1.389 (4)	C9C—C8C	1.368 (4)
C6B—H6B	0.9300	C9C—H9C	0.9300
C4C—O1C	1.236 (3)	C12A—H12A	0.9600
C4C—C2C	1.439 (4)	C12A—H12B	0.9600
C4C—C5C	1.503 (4)	C12A—H12C	0.9600
C6C—C7C	1.375 (4)	C10A—C9A	1.395 (4)
C6C—C5C	1.386 (4)	C12B—H12D	0.9600
C6C—H6C	0.9300	C12B—H12E	0.9600
O1A—C4A	1.235 (3)	C12B—H12F	0.9600
N1—C3B	1.320 (4)	C7A—C8A	1.381 (4)
N1—C11B	1.445 (4)	C7A—Cl1A	1.742 (3)
N1—C12B	1.458 (4)	C1B—H1B1	0.9700
C5B—C10B	1.404 (4)	C1B—H1B2	0.9700
C5B—C4B	1.503 (4)	C12C—H12G	0.9600
C2C—C3C	1.376 (4)	C12C—H12H	0.9600
C2C—C1C	1.500 (4)	C12C—H12I	0.9600
C5C—C10C	1.402 (4)	C8C—C7C	1.373 (4)
C2B—C3B	1.365 (4)	C8C—H8C	0.9300
C2B—C4B	1.447 (4)	C9A—C8A	1.371 (4)
C2B—C1B	1.501 (4)	C9A—H9A	0.9300
N2—C3A	1.321 (3)	C8A—H8A	0.9300
N2—C11A	1.454 (4)	C1C—H1C1	0.9700
N2—C12A	1.462 (3)	C1C—H1C2	0.9700
C6A—C7A	1.373 (4)	C11B—H11A	0.9600
C6A—C5A	1.386 (4)	C11B—H11B	0.9600
C6A—H6A	0.9300	C11B—H11C	0.9600
C3B—H3B	0.9300	C1A—H1A1	0.9700
N3—C3C	1.324 (3)	C1A—H1A2	0.9700
N3—C11C	1.450 (4)	C11A—H11D	0.9600
N3—C12C	1.460 (4)	C11A—H11E	0.9600
C5A—C10A	1.393 (4)	C11A—H11F	0.9600
C5A—C4A	1.513 (4)	C11C—H11G	0.9600
C9B—C8B	1.368 (4)	C11C—H11H	0.9600
C9B—C10B	1.394 (4)	C11C—H11I	0.9600
C9B—H9B	0.9300		
			
C10C—S1C—C1C	97.97 (14)	H12A—C12A—H12B	109.5
C10B—S1B—C1B	97.68 (15)	N2—C12A—H12C	109.5
C10A—S1A—C1A	97.13 (14)	H12A—C12A—H12C	109.5
C7B—C6B—C5B	120.7 (3)	H12B—C12A—H12C	109.5
C7B—C6B—H6B	119.6	C5A—C10A—C9A	120.0 (3)
C5B—C6B—H6B	119.6	C5A—C10A—S1A	120.7 (2)
O1C—C4C—C2C	123.7 (3)	C9A—C10A—S1A	119.3 (2)
O1C—C4C—C5C	117.0 (3)	N1—C12B—H12D	109.5
C2C—C4C—C5C	119.3 (2)	N1—C12B—H12E	109.5
C7C—C6C—C5C	120.3 (3)	H12D—C12B—H12E	109.5
C7C—C6C—H6C	119.8	N1—C12B—H12F	109.5
C5C—C6C—H6C	119.8	H12D—C12B—H12F	109.5
C3B—N1—C11B	124.6 (3)	H12E—C12B—H12F	109.5
C3B—N1—C12B	120.4 (3)	C6A—C7A—C8A	121.1 (3)
C11B—N1—C12B	115.0 (3)	C6A—C7A—Cl1A	119.2 (2)
C6B—C5B—C10B	118.6 (3)	C8A—C7A—Cl1A	119.7 (2)
C6B—C5B—C4B	118.0 (3)	C2B—C1B—S1B	112.7 (2)
C10B—C5B—C4B	123.3 (3)	C2B—C1B—H1B1	109.1
C3C—C2C—C4C	115.2 (2)	S1B—C1B—H1B1	109.1
C3C—C2C—C1C	126.7 (3)	C2B—C1B—H1B2	109.1
C4C—C2C—C1C	118.0 (2)	S1B—C1B—H1B2	109.1
C6C—C5C—C10C	119.3 (3)	H1B1—C1B—H1B2	107.8
C6C—C5C—C4C	117.8 (2)	N3—C12C—H12G	109.5
C10C—C5C—C4C	122.7 (2)	N3—C12C—H12H	109.5
C3B—C2B—C4B	115.5 (3)	H12G—C12C—H12H	109.5
C3B—C2B—C1B	126.1 (3)	N3—C12C—H12I	109.5
C4B—C2B—C1B	118.4 (3)	H12G—C12C—H12I	109.5
C3A—N2—C11A	125.8 (3)	H12H—C12C—H12I	109.5
C3A—N2—C12A	120.2 (2)	C9C—C8C—C7C	119.5 (3)
C11A—N2—C12A	114.0 (2)	C9C—C8C—H8C	120.2
C7A—C6A—C5A	120.6 (3)	C7C—C8C—H8C	120.2
C7A—C6A—H6A	119.7	C8A—C9A—C10A	120.8 (3)
C5A—C6A—H6A	119.7	C8A—C9A—H9A	119.6
N1—C3B—C2B	132.3 (3)	C10A—C9A—H9A	119.6
N1—C3B—H3B	113.9	C9A—C8A—C7A	118.9 (3)
C2B—C3B—H3B	113.9	C9A—C8A—H8A	120.5
C3C—N3—C11C	124.4 (3)	C7A—C8A—H8A	120.5
C3C—N3—C12C	119.6 (3)	C6B—C7B—C8B	120.9 (3)
C11C—N3—C12C	115.5 (3)	C6B—C7B—Cl1B	119.2 (3)
C6A—C5A—C10A	118.6 (3)	C8B—C7B—Cl1B	119.9 (3)
C6A—C5A—C4A	117.9 (2)	C2C—C1C—S1C	112.4 (2)
C10A—C5A—C4A	123.4 (3)	C2C—C1C—H1C1	109.1
C8B—C9B—C10B	120.8 (3)	S1C—C1C—H1C1	109.1
C8B—C9B—H9B	119.6	C2C—C1C—H1C2	109.1
C10B—C9B—H9B	119.6	S1C—C1C—H1C2	109.1
C9C—C10C—C5C	118.7 (3)	H1C1—C1C—H1C2	107.9
C9C—C10C—S1C	119.3 (2)	C8C—C7C—C6C	120.9 (3)
C5C—C10C—S1C	121.9 (2)	C8C—C7C—Cl1C	119.7 (2)
N3—C3C—C2C	132.0 (3)	C6C—C7C—Cl1C	119.3 (3)
N3—C3C—H3C	114.0	N1—C11B—H11A	109.5
C2C—C3C—H3C	114.0	N1—C11B—H11B	109.5
N2—C3A—C2A	133.1 (3)	H11A—C11B—H11B	109.5
N2—C3A—H3A	113.4	N1—C11B—H11C	109.5
C2A—C3A—H3A	113.4	H11A—C11B—H11C	109.5
O1A—C4A—C2A	124.6 (3)	H11B—C11B—H11C	109.5
O1A—C4A—C5A	116.4 (3)	C2A—C1A—S1A	112.9 (2)
C2A—C4A—C5A	119.0 (2)	C2A—C1A—H1A1	109.0
C3A—C2A—C4A	115.7 (2)	S1A—C1A—H1A1	109.0
C3A—C2A—C1A	126.1 (3)	C2A—C1A—H1A2	109.0
C4A—C2A—C1A	118.2 (2)	S1A—C1A—H1A2	109.0
C9B—C10B—C5B	119.5 (3)	H1A1—C1A—H1A2	107.8
C9B—C10B—S1B	119.4 (2)	N2—C11A—H11D	109.5
C5B—C10B—S1B	120.9 (2)	N2—C11A—H11E	109.5
C9B—C8B—C7B	119.4 (3)	H11D—C11A—H11E	109.5
C9B—C8B—H8B	120.3	N2—C11A—H11F	109.5
C7B—C8B—H8B	120.3	H11D—C11A—H11F	109.5
O1B—C4B—C2B	123.6 (3)	H11E—C11A—H11F	109.5
O1B—C4B—C5B	117.0 (3)	N3—C11C—H11G	109.5
C2B—C4B—C5B	119.4 (3)	N3—C11C—H11H	109.5
C8C—C9C—C10C	121.1 (3)	H11G—C11C—H11H	109.5
C8C—C9C—H9C	119.5	N3—C11C—H11I	109.5
C10C—C9C—H9C	119.5	H11G—C11C—H11I	109.5
N2—C12A—H12A	109.5	H11H—C11C—H11I	109.5
N2—C12A—H12B	109.5		
			
C7B—C6B—C5B—C10B	1.5 (4)	C4B—C5B—C10B—S1B	−1.3 (4)
C7B—C6B—C5B—C4B	−174.7 (3)	C1B—S1B—C10B—C9B	150.2 (3)
O1C—C4C—C2C—C3C	−11.4 (4)	C1B—S1B—C10B—C5B	−33.6 (3)
C5C—C4C—C2C—C3C	167.4 (3)	C10B—C9B—C8B—C7B	0.0 (5)
O1C—C4C—C2C—C1C	171.2 (3)	C3B—C2B—C4B—O1B	6.2 (5)
C5C—C4C—C2C—C1C	−10.0 (4)	C1B—C2B—C4B—O1B	−175.4 (3)
C7C—C6C—C5C—C10C	−2.8 (4)	C3B—C2B—C4B—C5B	−172.9 (3)
C7C—C6C—C5C—C4C	171.9 (3)	C1B—C2B—C4B—C5B	5.6 (4)
O1C—C4C—C5C—C6C	−17.8 (4)	C6B—C5B—C4B—O1B	18.5 (5)
C2C—C4C—C5C—C6C	163.3 (3)	C10B—C5B—C4B—O1B	−157.6 (3)
O1C—C4C—C5C—C10C	156.7 (3)	C6B—C5B—C4B—C2B	−162.5 (3)
C2C—C4C—C5C—C10C	−22.2 (4)	C10B—C5B—C4B—C2B	21.5 (5)
C11B—N1—C3B—C2B	10.6 (6)	C5C—C10C—C9C—C8C	−3.1 (5)
C12B—N1—C3B—C2B	−172.9 (3)	S1C—C10C—C9C—C8C	179.3 (3)
C4B—C2B—C3B—N1	−172.3 (3)	C6A—C5A—C10A—C9A	1.4 (4)
C1B—C2B—C3B—N1	9.4 (6)	C4A—C5A—C10A—C9A	−173.9 (3)
C7A—C6A—C5A—C10A	−0.6 (4)	C6A—C5A—C10A—S1A	177.9 (2)
C7A—C6A—C5A—C4A	175.0 (3)	C4A—C5A—C10A—S1A	2.6 (4)
C6C—C5C—C10C—C9C	4.6 (4)	C1A—S1A—C10A—C5A	33.8 (3)
C4C—C5C—C10C—C9C	−169.9 (3)	C1A—S1A—C10A—C9A	−149.7 (3)
C6C—C5C—C10C—S1C	−177.9 (2)	C5A—C6A—C7A—C8A	0.0 (5)
C4C—C5C—C10C—S1C	7.7 (4)	C5A—C6A—C7A—Cl1A	−179.4 (2)
C1C—S1C—C10C—C9C	−154.8 (2)	C3B—C2B—C1B—S1B	131.1 (3)
C1C—S1C—C10C—C5C	27.7 (3)	C4B—C2B—C1B—S1B	−47.1 (4)
C11C—N3—C3C—C2C	−14.4 (5)	C10B—S1B—C1B—C2B	56.2 (3)
C12C—N3—C3C—C2C	174.5 (3)	C10C—C9C—C8C—C7C	−0.1 (5)
C4C—C2C—C3C—N3	171.5 (3)	C5A—C10A—C9A—C8A	−1.6 (4)
C1C—C2C—C3C—N3	−11.3 (6)	S1A—C10A—C9A—C8A	−178.2 (2)
C11A—N2—C3A—C2A	−6.0 (6)	C10A—C9A—C8A—C7A	1.0 (5)
C12A—N2—C3A—C2A	174.9 (3)	C6A—C7A—C8A—C9A	−0.2 (5)
C6A—C5A—C4A—O1A	−19.5 (4)	Cl1A—C7A—C8A—C9A	179.2 (2)
C10A—C5A—C4A—O1A	155.8 (3)	C5B—C6B—C7B—C8B	−1.2 (5)
C6A—C5A—C4A—C2A	160.3 (3)	C5B—C6B—C7B—Cl1B	178.4 (2)
C10A—C5A—C4A—C2A	−24.4 (4)	C9B—C8B—C7B—C6B	0.4 (5)
N2—C3A—C2A—C4A	173.4 (3)	C9B—C8B—C7B—Cl1B	−179.2 (2)
N2—C3A—C2A—C1A	−7.3 (6)	C3C—C2C—C1C—S1C	−126.0 (3)
O1A—C4A—C2A—C3A	−4.2 (5)	C4C—C2C—C1C—S1C	51.1 (3)
C5A—C4A—C2A—C3A	176.0 (3)	C10C—S1C—C1C—C2C	−55.0 (2)
O1A—C4A—C2A—C1A	176.5 (3)	C9C—C8C—C7C—C6C	2.0 (5)
C5A—C4A—C2A—C1A	−3.2 (4)	C9C—C8C—C7C—Cl1C	−179.7 (2)
C8B—C9B—C10B—C5B	0.3 (5)	C5C—C6C—C7C—C8C	−0.5 (5)
C8B—C9B—C10B—S1B	176.6 (2)	C5C—C6C—C7C—Cl1C	−178.8 (2)
C6B—C5B—C10B—C9B	−1.0 (4)	C3A—C2A—C1A—S1A	−132.5 (3)
C4B—C5B—C10B—C9B	175.0 (3)	C4A—C2A—C1A—S1A	46.7 (4)
C6B—C5B—C10B—S1B	−177.3 (2)	C10A—S1A—C1A—C2A	−57.1 (3)

### Hydrogen-bond geometry (Å, º)

**Table:**

*D*—H···*A*	*D*—H	H···*A*	*D*···*A*	*D*—H···*A*
C1a—H1a2···O1b^i^	0.97	2.39	3.235 (4)	144
C11a—H11d···O1b^i^	0.96	2.63	3.290 (4)	126
C1b—H1b2···O1a	0.97	2.43	3.284 (1)	147
C12a—H12b···O1b	0.96	2.59	3.358 (4)	137

Symmetry code: (i) *x*, *y*−1, *z*.
